# Assessment of the Safety of a Combined Medicinal Agent Based on Probiotic Lactic Acid Bacteria and the Natural Component *Glycyrrhiza glabra*

**DOI:** 10.3390/biomedicines14102364

**Published:** 2026-10-16

**Authors:** Irina Ratnikova, Amankeldi Sadanov, Baiken Baimakhanova, Erik Shorabaev, Saltanat Orazymbet, Elmira Namaz, Nurshad Utegenova

**Affiliations:** 1 LLP «Industrial Microbiology», Al-Farabi Ave 89/44, Almaty 050040, Kazakhstan; iratnikova@list.ru (I.R.);; 2 Research and Production Center of Microbiology and Virology, Bogenbay Batyr St., 105, Almaty 050010, Kazakhstan; 3 Department of Botany and Agroecology, Al-Farabi Kazakh National University, Al-Farabi Ave 71, Almaty 050040, Kazakhstan

**Keywords:** natural products, medicinal plants, probiotics, toxicity, safety of medicinal agents

## Abstract

Natural products, including medicinal plants, are an important source of medicines. However, the natural origin does not exclude undesirable effects with prolonged use, which requires a systematic assessment of their safety. **Background/Objectives**: The aim of this work is to evaluate the safety of an antituberculous medicinal agent based on the probiotic association of lactic acid bacteria *Lacticaseibacillus rhamnosus* 7n, *Lacticaseibacillus paracasei* B-3/43, *Lacticaseibacillus casei* 14d/13 and 1% licorice extract (*Glycyrrhiza glabra*) with repeated administration. **Methods**: The experimental study was conducted on laboratory mice in accordance with a 60-day oral administration protocol at doses of 25 mg/kg, 250 mg/kg, and 2500 mg/kg body weight. The control group of animals received saline solution. The analysis included hematological, biochemical parameters, and a study of urine composition, as well as macro- and histological examinations of organs. The work was performed in accordance with the basic principles of GLP. **Results**: The agent did not cause general toxic effects, behavioral disorders and changes in feed intake were not noted. Morphofunctional changes were not detected. Macroscopic examination revealed no pathological changes in the internal organs. Histological analysis confirmed the absence of signs of degeneration and necrosis in the tissues of organs. Hematological parameters were within the limits of physiological norms. Blood biochemical parameters showed no significant differences compared to the control group. The parameters of the general urinalysis were without significant changes between the groups and gender. **Conclusions**: The medicinal agent showed a favorable safety profile without signs of toxicity.

## 1. Introduction

Currently, there is an increased interest in biologically active substances of natural origin, including medicinal plants and probiotic microorganisms. Their potential value is related to the variety of pharmacological effects. Special attention in this regard should be paid to licorice glabra (*Glycyrrhiza glabra* L.), the root of which has a wide range of pharmacological properties, including antibacterial, antiviral, anti-inflammatory, antioxidant and expectorant effects. Therefore, licorice extract and probiotic lactic acid bacteria can be included in the composition of a combined medicinal agent for the treatment of infectious diseases, including tuberculosis.

Tuberculosis (TB) is a global health problem and one of the leading causes of death worldwide. It is estimated that a quarter of the world’s population is infected with *Mycobacterium tuberculosis* (Mtb), and 5–10% of those infected develop tuberculosis during their lifetime [1]. Tuberculosis can be treated, prevented, and cured. The rapid and steady decline in tuberculosis-related deaths in many countries over the past 50 years is convincing evidence that ending the pandemic is possible. Nevertheless, this disease—which has plagued humanity for centuries and claims the lives of more than millions of people every year—remains an unrelenting scourge [2,3,4,5].

The current treatment regimen for drug-sensitive Mtb strains lasts 6 months and consists of simultaneous administration of several different medications that require strict adherence to prevent relapse and resistance. Poverty, lack of proper treatment, and difficulties in following the treatment instructions on the part of patients have contributed to the emergence of more dangerous drug-resistant strains that require even longer treatment with toxic and expensive drugs compared to the first regimen [6,7]. In this regard, the task of further searching and developing new approaches to the treatment and prevention of tuberculosis, combining such qualities as high efficiency against the causative agent of the disease and a minimum number of side effects, is urgent.

One of the promising directions in this context is the use of probiotic microorganisms. The definition of probiotics, which is widely known today, was formulated in 2001 by the Food and Agriculture Organization of the United Nations and the World Health Organization (FAO/WHO): “Living microorganisms that, when administered in sufficient quantities, benefit the health of the host [8,9].” The composition of probiotic products usually includes one or more strains of microorganisms. The main genera of probiotic microorganisms include *Lactobacillus*, *Bifidobacterium*, *Lactococcus*, *Streptococcus*, and *Enterococcus*. The main principles of action of probiotics, justifying the prospects of their use in the treatment and prevention of tuberculosis, are: (i) antagonistic action provided by the synthesis of antimicrobial compounds; (ii) competition with pathogenic organisms for both epithelial adhesion and vital nutrients; (iii) modulation of innate and adaptive immune responses of the host; and (iv) suppression of bacterial toxin production [10,11]. Probiotic strains belonging to the lactic acid bacteria group (Lactic Acid Bacteria, LAB) officially have the status of “Generally Recognized as Safe” (GRAS—“Generally Recognized as Safe”) and are widely used in microbiology due to the following properties: immunomodulatory, antioxidant, antimicrobial and anti-inflammatory ability, maintenance of the barrier function of mucous membranes and regulation of the composition of the microbiota [12,13,14,15,16,17].

The new antituberculosis combination of medicinal agent was created as part of the scientific and technical project “Preclinical trials of a domestic original drug against human tuberculosis” (2024–2026). In the composition of this medicinal agent, along with lactic acid bacteria *Lacticaseibacillus rhamnosus* 7n, *Lacticaseibacillus parasasei* B-3/43, *Lacticaseibacillus casei* 14d/13, an extract of the medicinal plant licorice *Glycyrrhiza glabra* was added. The roots of this plant are traditionally used to treat chronic inflammatory diseases. Due to the presence of glycyrrhizic acid and glycyrrhetinic acid, licorice roots have a wide range of pharmacological properties, including antibacterial, antiviral, anti-inflammatory, antiarrhythmic, antioxidant, and expectorant effects [18,19,20,21,22]. In addition, there is evidence in the literature about the potential prebiotic properties of *Glycyrrhiza glabra*. In an in vitro study using anaerobic cultures of human feces, it was found that *Glycyrrhiza glabra* powder is able to change the composition of the intestinal microbial community, in favor of increasing the number of beneficial bacterial taxa [23]. The prebiotic properties of the alcoholic extract of licorice root used in the composition of the studied medicinal product have not been studied separately and require additional study. The composition of the investigated agent was selected based on a number of studies conducted by us earlier. So far, there have been no reports of probiotics that are reliably active against mycobacteria, and there are only isolated studies in this field. According to the literature, there is also no information on the use of probiotic bacteria in combination with extracts of medicinal plants for the treatment of tuberculosis infection.

Despite the fact that the components included in the investigational medicinal agent (lactic acid bacteria strains and licorice extract) are characterized by a favorable safety profile, rare undesirable effects have been described in the literature, mainly associated with prolonged use or high doses. In particular, the use of licorice-containing drugs can lead to pseudohyperaldosteronism, manifested by hypokalemia, increased blood pressure, fluid retention and edema; in severe cases, muscular and cardiovascular complications are described. The description of the use of lactic acid bacteria also describes isolated cases of bacteremia and septic complications, mainly found in patients with severe concomitant diseases or immune disorders. Data on the effects of prolonged use of probiotic associations in medicinal products, especially in combination with plant extracts, remain limited and require experimental confirmation. Therefore, the development of a medicinal agent based on these components requires a comprehensive preclinical safety assessment, including the study of acute, subacute and chronic toxicity. Only after a comprehensive assessment of safety and efficacy can we proceed to clinical trials, as well as the further introduction into pharmacology [24,25,26,27,28].

The aim of the study is to study the safety of a medicinal agent based on natural components, namely the association of probiotic lactic acid bacteria *Lacticaseibacillus rhamnosus* 7n, *Lacticaseibacillus parasasei* B-3/43, *Lacticaseibacillus casei* 14d/13 and licorice extract *Glycyrrhiza glabra* with 60-day oral administration.

## 2. Materials and Methods

### 2.1. Investigated Medicinal Agent and Test System

The object of research was a medicinal agent based on the association of probiotic lactic acid bacteria *Lacticaseibacillus rhamnosus* 7n, *Lacticaseibacillus parasasei* B-3/43, *Lacticaseibacillus casei* 14d/13 and licorice extract (*Glycyrrhiza glabra*). The agent is an opaque liquid with a characteristic sour-milk odor. The main components of this agent are probiotic lactic acid bacteria in the amount of 8.5 × 10^9^ CFU/mL and 1% alcohol extract of licorice. The experimental study was conducted on laboratory mice in accordance with a 60-day protocol for oral administration of the investigated agent at doses of 25.0, 250.0 and 2500.0 mg/kg body weight.

The medicinal product must be stored in a dry place protected from direct light, at a temperature from +2 °C to +8 °C, and the shelf life under these conditions is up to 5 months. When using a protector containing 7% sucrose and 1.5% gelatin, the shelf life increases to 9 months. At the same time, the initial amount of lactic acid bacteria was 8.5 × 10^9^ CFU/mL, by the end of the shelf life—2.0 × 10^9^ CFU/mL.

The study was conducted in accordance with the documents [29,30].

### 2.2. Preparation of the Investigational Medicinal Agent

Medicinal plant raw materials—roots of *Glycyrrhiza glabra* L. (batch N. 0424)—were purchased from the pharmacy chain of the Republic of Kazakhstan and certified for compliance with the requirements of the State Pharmacopeia of the RK. Before extraction, the plant raw materials were cleaned of impurities and crushed to a particle size of 3–5 mm. The licorice extract was prepared by ultrasonic extraction using an ultrasonic probe (KFS-600 N, Coprotech, Seoul, Republic of Korea) [31]. After preliminary maceration, the samples were extracted using ultrasound at a frequency of 45 kHz, a temperature of 35–45 °C and an extraction time of 35 min. The extraction was carried out at a ratio of 1:10 of plant raw materials and extractant. For example, 100 mL of extractant was used for 10 g of crushed vegetable raw materials. The liquid licorice root extract was obtained by ultrasonic extraction using purified water and 50% (*v*/*v*) aqueous ethanol as extractants. Purified water complied with the requirements of the State Pharmacopoeia of the Republic of Kazakhstan (SP RK, Volume II). Ethanol (96%, pharmaceutical grade) also complied with the requirements of the SP RK (Volume II). The use of hydroethanol solutions above 50% was not considered necessary, since the goal was to obtain liquid extracts potentially compatible with the probiotic component. The choice of hydroethanol solvents was determined by the chemical composition of licorice root, which contains compounds of various polarities, including glycyrrhizic acid, triterpene saponins, flavonoids, phenolic compounds, sugars and other water-soluble and alcohol-soluble components. Water helps to extract more polar compounds, while the presence of ethanol improves the intake of moderately polar compounds into the extract, primarily flavonoids and some phenolic components.

The study used three strains of lactic acid bacteria *Lacticaseibacillus rhamnosus* 7n, *Lacticaseibacillus parasasei* B-3/43, *Lacticaseibacillus casei* 14d/13, previously isolated from the intestine of a healthy person in the laboratory of microbial preparations of Industrial Microbiology LLP, which are stored in the RCM under the numbers B-RKM 1280 (*Lacticaseibacillus rhamnosus* 7n), B-RKM 1289 (*Lacticaseibacillus parasasei* B-3/43), B-RKM 1284 (*Lacticaseibacillus casei* 14d/13).

Each of the bacterial strains was grown separately on an MRS liquid medium (HiMedia Laboratories Pvt. Ltd., Mumbai, India) for 24 h at a temperature of 37 °C.

Three bacterial strains were combined into an association in a ratio of 1:1:1. The resulting association in a volume of 0.5% was seeded into a liquid MRS medium and cultured for 24 h at a temperature of 37 °C.

Water–alcohol extracts of a medicinal plant were added to test tubes with a grown probiotic association. To assess the effect of the extract concentration on the properties of the probiotic association, samples containing plant extracts in concentrations of 2.5%, 1.0% and 0.7% were prepared. As a result, experimental samples were obtained: a probiotic association, a separate plant extract, and a combination of “association + plant extract” variants.

Next, antimicrobial activity was determined by diffusion into agar. Antimicrobial activity is an important indicator in the development of a combined dosage form of the “plant extract–probiotic” type. Antimicrobial activity characterizes the ability of plant extracts to directly inhibit the growth of the tested microorganisms due to the biologically active compounds contained in them.

Antimicrobial activity, in turn, reflects the ability of probiotic lactic acid bacteria and their combinations with plant extracts to inhibit the growth of the tested microorganism (*Mycobacterium* B-5). The evaluation of these indicators makes it possible to determine both the independent effect of plant components and their effect on the biological activity of the probiotic association. An equal volume of each test sample was added to each well. Petri dishes were incubated under conditions optimal for the growth of mycobacteria. In the presence of inhibitory activity, a zone of lack of growth or pronounced suppression of the growth of the tested microorganisms formed around the well. After incubation, the diameters of the growth inhibition zones around the wells were measured in millimeters. The data obtained made it possible to compare and determine the activity of the association of probiotics, a separate plant extract and a combined drug.

*Mycobacterium* B-5 growth suppression zones: Bacterial association—30 mm, association + licorice extract (0.7%)—32 mm, association + licorice extract (1%)—40 mm, association + licorice extract (2.5%)—25 mm. The number of viable cells in the association variants is 8.5 × 10^9^ CFU/mL, the association + licorice extract (0.7%) is 8.5 × 10^9^ CFU/mL, the association + licorice extract (1%) is 8.5 × 10^9^ CFU/mL, the association + licorice extract (2.5%) is 6.0 × 10^9^ CFU/mL. At the same time, the optimal concentration of licorice extract (1%) is maintained, while maintaining the high viability of probiotic cells and simultaneously enhancing the antimicrobial activity of the probiotic association. This concentration is more favorable for maintaining the viability of the probiotic association compared to the 2.5% option. It is likely that at a concentration of 1.0%, biologically active compounds of licorice extract do not have a pronounced inhibitory effect on lactic acid bacteria and allow them to maintain a high number of their cells.

### 2.3. Conditions of Detention, Care and Control of Test Systems

The study used outbred white mice weighing 22.0 g ± 20%. The animals were divided into three experimental groups depending on the administered dose of the agent (group 1–3), a control group (group 4) and an intact control group (group 5). Each group contained 20 individuals of both sexes for each dose studied (10 ♂ and 10 ♀), and for the control groups (10 ♂ and 10 ♀).

The pre-experimental veterinary examination of the animals was conducted in accordance with SOP-PHT-011 Veterinary Animal Control.

The quarantine of animals was 12 days in a room for keeping laboratory animals. After at least 7 days of quarantine to assess their health (pre-experimental veterinary clearance), the selected animals underwent an additional 5-day acclimatization period to adapt to the experimental conditions. The total duration of the adaptation period was at least 12 calendar days (7 days of quarantine + 5 days of acclimatization).

The animals for research were weighed before the start of the experiment and 10 individuals were placed in a cage.

The animals were kept at a temperature of 22 ± 3 °C, a relative humidity of 50–60% and a 12 h light regime with free access to water and food.

Environmental conditions, nutrition, water, body weight control, veterinary control and medical examination were monitored according to:

SOP-PHT-001 Laboratory Animal Care/Care of laboratory animals;

SOP-PHT-011 Veterinary Care/Veterinary control;

SOP-PHT-022 Feed Storage and Feeding For Laboratory Animals;

SOP-PHT-021 Animal Observation.

Identification/labeling of each animal was carried out according to

SOP-PHT-004. The complete list of standard operating procedures (SOPs) used in this study is provided in the Supplementary Materials.

### 2.4. Administration of the Investigational Medicinal Agent to Mice

Working solutions of the studied agent:

- 1–3 groups: doses of 25.0 mg/kg; 250.0 mg/kg; 2500.0 mg/kg were used. The initial solution of the drug was used to prepare a maximum dose of 2500 mg/kg. Working solutions for the 25 and 250 mg/kg dose groups were prepared by sequential dilution of the initial preparation with sterile saline. The 2500 mg/kg dose was administered using the initial preparation, as specified in the study protocol. The administration volume was 0.5 mL per animal in all dose groups. The working solutions were prepared and administered at the Scientific Center for Anti-Infectious Drugs in accordance with the approved study protocol. The dose calculations were based on the animal body weights and the concentrations specified in the protocol and the applicable principles of good laboratory practice (GLP).
- Group 4: Each animal was given 0.5 mL of ready-made saline solution per day.
- Group 5: Intact control.

The investigational agent was administered orally for 60 days.

### 2.5. Integral Tests: Examination, General Behavior, Somatic Parameters, Survival, Weight Gain, Food Intake

All animals were observed during the entire period of administration (60 days) of solutions of the medicinal agent. During the follow-up period, the presence of signs of toxic effects associated with exposure to the study agent, survival and general behavior were assessed.

After 60 days of administration/observation, the animals were euthanized, autopsied, macroscopied, and the masses of the following organs were measured: heart, liver, spleen, kidneys, lung, testicles, and ovaries.

To determine the degree of food intake, feeders with and without food were weighed before feeding the mice. Calculations of feed consumption were carried out weekly.

Analysis of somatic parameters. Changes in the external somatic signs of pathological administration (behavior, condition of the coat, coloration of the oral mucosa, and other signs) were carried out during daily monitoring of laboratory animals. The observation parameters, as well as signs of their changes, were recorded in the primary data sheets.

The monitoring parameters, as well as the signs of their change, are described in SOP-PHT-021.

Analysis of weight characteristics. The animals were weighed once a week, starting from the first day of the study. Body weight was measured in grams.

After the autopsy, organs (lungs, heart, liver, spleen, kidneys, testicles or ovaries) were weighed, and relative weight coefficients were calculated.

Feed consumption. To determine the degree of food intake, feeders with and without food were weighed before feeding. Calculations of feed consumption were carried out based on the difference in the weights of the feeder before feeding the mice and after 7 days.

### 2.6. Euthanasia of Animals

The animals were removed from the experiment on the 60th day after the administration of doses of the studied agents.

Mice were removed from the experiment by cervical dislocation after isoflurane anesthesia with SOP-PHT-016.

### 2.7. Macroscopic Analysis

After stopping the administration of the test substance on day 60, the animals were subjected to autopsy and macroscopic description.

Macroscopic analysis was performed according to SOP-PHT-019.

### 2.8. Hematological Studies

Hematological examinations were performed in all groups at the end of the experiment.

The study identified the main peripheral blood parameters characterizing the state of the hematopoiesis system, including the number of red blood cells (RBC), hemoglobin (HGB), hematocrit (HCT), the number of leukocytes (WBC), platelets (PLT), as well as indicators of the leukocyte formula: lymphocytes, neutrophils, monocytes, eosinophils and basophils.

### 2.9. Biochemical Research

Biochemical studies were carried out in all groups at the end of the experiment.

In a biochemical study of blood serum, the following parameters were studied: aspartate transaminase, alanine transaminase bilirubin, total protein, urea, creatinine, total cholesterol, alkaline phosphatase.

### 2.10. Urine Testing

The urine sediment was examined in all groups at the end of the experiment. The following parameters were studied: protein, bilirubin, ketone bodies, urobilinogen, urea, sodium, potassium ions, urine sediment analysis.

### 2.11. Histology of Organs

Animals of the control group and the group exposed to the maximum dose of the test substance are subject to histological examination; organs of animals of other groups are histologically examined as necessary, when macroscopic changes in organs are detected, when target organs are identified during slaughter.

The following organs and tissues of animals are subject to histological examination: lymph nodes, thymus, lungs, heart, liver, spleen, kidneys, adrenal glands, testicles, ovaries, brain, and other organs and tissues with macroscopic changes. 

Histological analysis was performed according to SOP-PHT-020 preparation of histological sections.

### 2.12. Criteria for Evaluating Test Results

The study of toxicity following repeated oral administration is aimed at determining the toxic effects of the tested medicinal agent and identifying potential target organs.

The evaluation of the research results was carried out based on the following criteria:

- Survival/mortality of animals during the entire period of administration/observation of 60 days;
- Somatic indicators;
- Body weight of the animals receiving the test solutions;
- Food consumption;
- Urine tests;
- Macroscopy of internal organs;
- Weight coefficients of internal organs;
- Hematological studies;
- Biochemical research;
- Histological studies.

### 2.13. Calculation of Results and Statistical Methods

All calculations will be performed using Microsoft Excel 2007. Standard statistical methods will be used to process the results. The standard deviation (SD) is calculated for all numerical data.

To assess the differences in animal weight between the experimental groups, a one-way analysis of variance (one-way ANOVA) was performed, followed by multiple comparisons using the Sidak criterion. The statistical significance of the differences was assessed at the significance level (*p* < 0.05).

## 3. Results

### 3.1. Observation of Experimental Animals

During the period of oral administration of the investigational medicinal agent in doses of 25.0, 250.0 and 2500.0 mg/kg, as well as during the subsequent follow-up period (the total duration of the study was 60 days), no signs of toxic effects were detected in animals.

The results of daily clinical observations showed no mortality in all experimental groups. During the entire study and recovery period, the animals showed no general health or behavior disorders, including uncoordinated movements, lacrimation, increased salivation or foaming, diarrhea, vomiting, piloerection, crowding of animals, prostration, tremor, difficulty breathing, hunched posture, decreased response to external stimuli, refusal of food, pallor of mucous membrane skin changes, seizures, and decreased skeletal muscle tone.

All animals maintained a satisfactory general condition throughout the study. On daily examination, normal motor activity, an adequate response to grabbing, touching, visual and auditory stimuli, as well as a sustained response to tail pinching were noted.

The results obtained indicate the absence of clinical signs of systemic toxicity and negative effects of the studied agent on the functional state of the central nervous system and behavioral reactions of animals with prolonged oral administration.

### 3.2. Analysis of Somatic and Weight Parameters

The dynamics of changes in animal body weight (average values) during the experiment are presented in Table 1, Table 2, Table 3 and Table 4.

All calculations will be performed using Microsoft Excel 2007. Standard statistical methods were used to process the results. The standard deviation (±SD) is calculated for all numerical data.

The significance of the differences between the groups is assessed using the *t*-test, with a significance level of *p* < 0.05.

A comparative analysis of body weight dynamics showed that in males and females who received the studied agent at doses of 25.0, 250.0 and 2500.0 mg/kg, body weight changes were comparable with those in the control group. There was no dose-dependent decrease in body weight gain or decrease in animal weight.

Slight fluctuations in the body weight of individual animals were of an individual nature and were within the limits of physiological variability. The results obtained indicate that there is no negative effect of the studied agent on the growth, development and general physiological condition of animals with repeated oral administration.

The calculation of the relative weights of the organs also revealed no deviations from the physiological values. The weight coefficients of the liver, kidneys, spleen, heart, lungs, and sex glands were comparable between the animals of the group and showed no signs of an increase or decrease in the mass of individual organs, which could indicate the development of toxic effects of the studied medicinal agent.

Data on the dynamics of body weight and its gain coefficient in male and female mice with 60-day oral administration of the studied agent are presented in Table 5 and Table 6, respectively.

The average animal weights were 21.31 in the 25 mg/kg group, 21.93 in the 250 mg/kg group, 21.66 in the 2500 mg/kg group, 21.65 in the control group, and 21.78 in the intact group. The highest average value was recorded in the 250 mg/kg group, the lowest in the 25 mg/kg group. The difference between the highest and lowest average values was 0.62 units of measurement (Table 7).

One-factor analysis of variance revealed no statistically significant differences between the mean values of the five groups: F(4, 5) = 1.907; *p* = 0.2476. The coefficient of determination R2 was 0.6041. Therefore, at the selected significance level, the data obtained do not provide grounds to assert the presence of statistically significant intergroup differences in animal weight (Table 8).

The results of pairwise comparisons with the Sidak correction are consistent with the general result of the ANOVA: no statistically significant differences were found between individual pairs of groups (all adjusted *p* > 0.05). The lowest adjusted *p*-value was obtained when comparing groups of 25 and 250 mg/kg (*p* = 0.3415), but it also exceeds the established significance level (Table 9).

There were no statistically significant differences in the average weight of animals between the groups in the presented sample (single-factor ANOVA: F(4, 5) = 1.907; *p* = 0.2476; pairwise comparisons according to the Sidak criterion—*p* > 0.05).

### 3.3. Feed Consumption Monitoring

During the 60-day follow-up period, animal feed intake in all experimental and control groups remained stable. The average values of daily feed intake were comparable between the groups and were within the limits of physiological variability. There were no statistically significant differences between the animals receiving the studied agent at doses of 25, 250 and 2500 mg/kg and the control group (*p* > 0.05). The data obtained indicate that there is no negative effect of the studied agent on the appetite and eating behavior of animals.

### 3.4. Results of Macroscopic Description of Organs

After 60 days of oral administration of the medicinal agent in the studied dosages, the animals were decapitated and macroscopied.

No pathological changes were found during macroscopic examination of animals after 60 days of oral administration of the investigational drug in doses of 25.0, 250.0 and 2500.0 mg/kg. The coat was clean, without areas of alopecia; the mucous membranes of the eyes, nose and mouth were pale pink, and there was no pathological discharge. The teeth were properly sharpened, the external auditory canals were clean, and the vibrissae were without any peculiarities. The skin showed no signs of inflammation, phlegmon, edema, hematomas and nodules of subcutaneous tissue. The location of the internal organs is typical for this type of animal. There was no free fluid in the thoracic and abdominal cavities. Inguinal, bronchial, axillary, cervical, mandibular, and mesenteric lymph nodes were not enlarged. No macroscopic abnormalities were found in the prostate gland, diaphragm, and thymus. The heart muscle is homogeneous, dark red, with no visible damage or hemorrhage; the aorta was well visualized. There was no thickening of the wall of the left ventricle during cross-section. The lung tissue retained its lightness. Upon dissection, light, slightly frothy contents were released from the large bronchi. The liver had a uniform dark cherry color; there were no signs of organ enlargement or venous fullness. The spleen was dark red, without hemorrhages or ulceration. The kidneys were characterized by a smooth, even surface and a dark brown color. On the sagittal section, the cortical and cerebral layers were clearly distinguished, while the cortical substance prevailed. The adrenal glands were rounded and cream-colored, located on top of the kidneys. The serous membranes of the stomach and intestines were smooth and shiny, while the Peyer’s patches remained unchanged. No necrosis foci or additional formations were found in the pancreas. The remains of food remained in the stomach without signs of a putrid odor; the folding of the mucous membrane was preserved and ulcerative defects were absent. In the duodenum, upon cross-section, the presence of a homogeneous yellow chyme was noted. The stool in the large intestine was well-formed. The macroscopic condition of the organs of the reproductive system corresponded to the norm. The uterine cavity was free, and the uterine horns were passable during probing. The brain had a typical light gray color and the vascular pattern was visualized; no pathological foci were found in the cranial cavity.

Autopsy and macroscopy of experimental animals after repeated oral administration of a sample of the agent in liquid form at doses of 25.0 mg/kg; 250.0 mg/kg; 2500.0 mg/kg for 60 days did not reveal significant/pronounced deviations. The arrangement of the internal organs is correct/typical, there was no evidence of any solderage, or a sharp increase or decrease in size.

### 3.5. Results of Hematological Studies

During hematological examination after 60 days of oral administration of the agent in three different doses, the following parameters were studied: erythrocytes (RBC), hemoglobin (HGB), hematocrit (HCT), white blood cell count (WBC), platelets (PLT), as well as indicators of the leukocyte formula: lymphocytes, neutrophils, monocytes, eosinophils and basophils.

After 60 days of oral administration of the investigational agent to male and female mice at doses of 25, 250 and 2500 mg/kg, no significant changes in the main hematological parameters were detected.

Males of 25 mg/kg in most hematological parameters practically did not differ from the control group. Minor fluctuations in the number of red blood cells, white blood cells, and individual populations of white blood cells were within the limits of physiological variability.

250 mg/kg also showed no significant changes. The mean values of RBC, HGB, HCT, WBC, and platelets were comparable to the control. The leukocyte formula retained the predominance of lymphocytes characteristic of mice.

2500 mg/kg was characterized by a slightly higher average number of red blood cells compared with the groups of 25 and 250 mg/kg; however, the increase was not accompanied by a corresponding increase in hemoglobin, hematocrit or other signs of hemoconcentration and was not dose-dependent.

The control group and intact animals had values comparable to those of the experimental groups. There were no significant differences between the control and intact animals.

Females of 25 mg/kg were characterized by slightly higher values of red blood cells compared with the control group; however, hemoglobin and hematocrit remained within physiological limits.

250 mg/kg practically did not differ from the control in all the main parameters.

2500 mg/kg had a slightly higher average white blood cell count compared to other groups of females; however, the increase was mainly due to physiological fluctuations in lymphocytes and was not accompanied by neutrophilic leukocytosis, which does not indicate an inflammatory reaction.

The control and intact groups showed similar values for most indicators.

### 3.6. The Results of Biochemical Research

Biochemical studies were carried out in all groups at the end of the experiment. The following parameters were studied in the biochemical study of blood serum: aspartate transaminase, alanine transaminase, total protein, urea, creatinine, total cholesterol, alkaline phosphatase, bilirubin.

Males of 25 mg/kg were characterized by values of AST, ALT, total bilirubin, urea, creatinine, total protein, alkaline phosphatase and cholesterol, which were within the limits of physiological variability.

250 mg/kg did not differ from the 25 mg/kg group and the control in most biochemical parameters. The activity of liver enzymes (AST and ALT) did not show a steady increase.

2500 mg/kg also showed no dose-dependent increase in AST, ALT, or bilirubin. Minor fluctuations in individual indicators were of an individual nature.

The control and intact groups had comparable values of all the studied indicators.

Thus, the males showed no signs of impaired liver function (AST, ALT, bilirubin), kidney function (urea and creatinine), or protein metabolism.

Females of 25 mg/kg were characterized by slightly greater individual variability in AST and ALT activity compared with males, but the average values did not indicate toxic liver damage.

250 mg/kg had a biochemical profile comparable to the control group. Concentrations of total protein, urea, creatinine, and cholesterol remained stable.

2500 mg/kg was not accompanied by a dose-dependent increase in liver enzymes or bilirubin concentration. The values of kidney function parameters also did not differ from the control.

The control and intact groups had similar biochemical parameters.

### 3.7. Urine Test Results

Urine tests were performed in all groups at slaughter. In the study of urine, the following parameters were studied: urine pH, protein, bilirubin, glucose, ketone bodies, urobilinogen, nitrites. Urine sediment analysis was performed. After 60 days of oral administration of the investigational agent in doses of 25, 250 and 2500 mg/kg; no pathological changes in the parameters of the general urinalysis were detected in the mice of both sexes.

### 3.8. The Results of Histological Studies

Histological studies were performed for a group of animals receiving a maximum dose of 2500.0 mg/kg of the studied medicinal agent.

**Figure 1:**
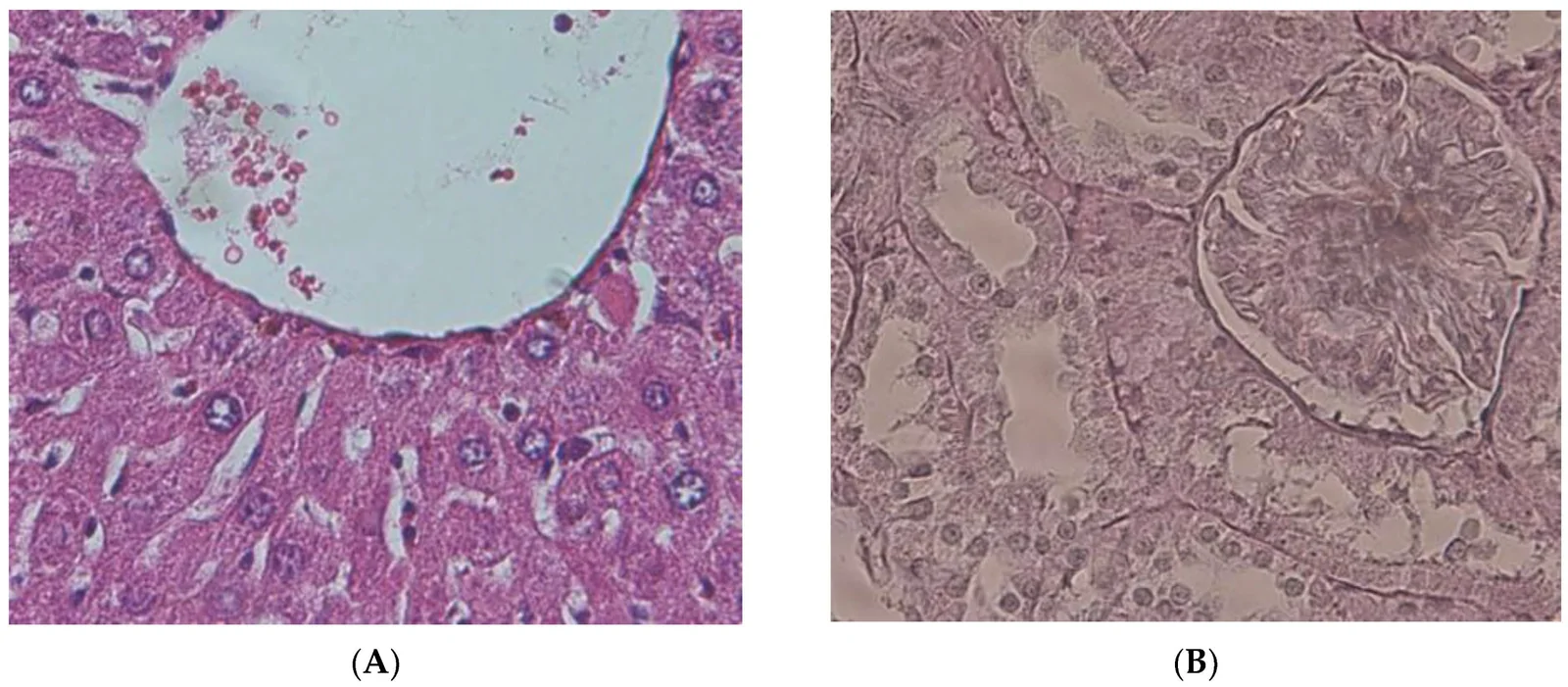

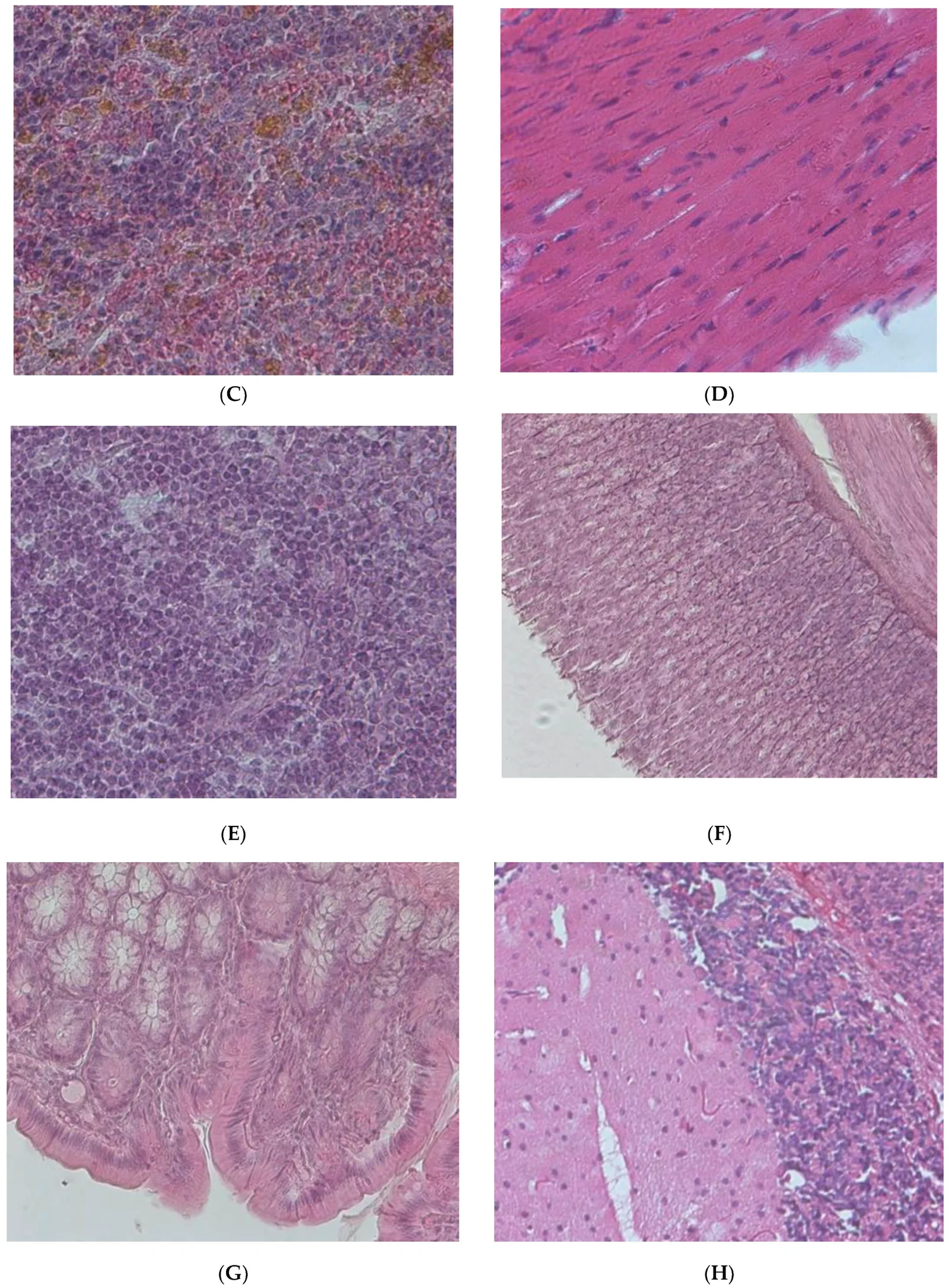

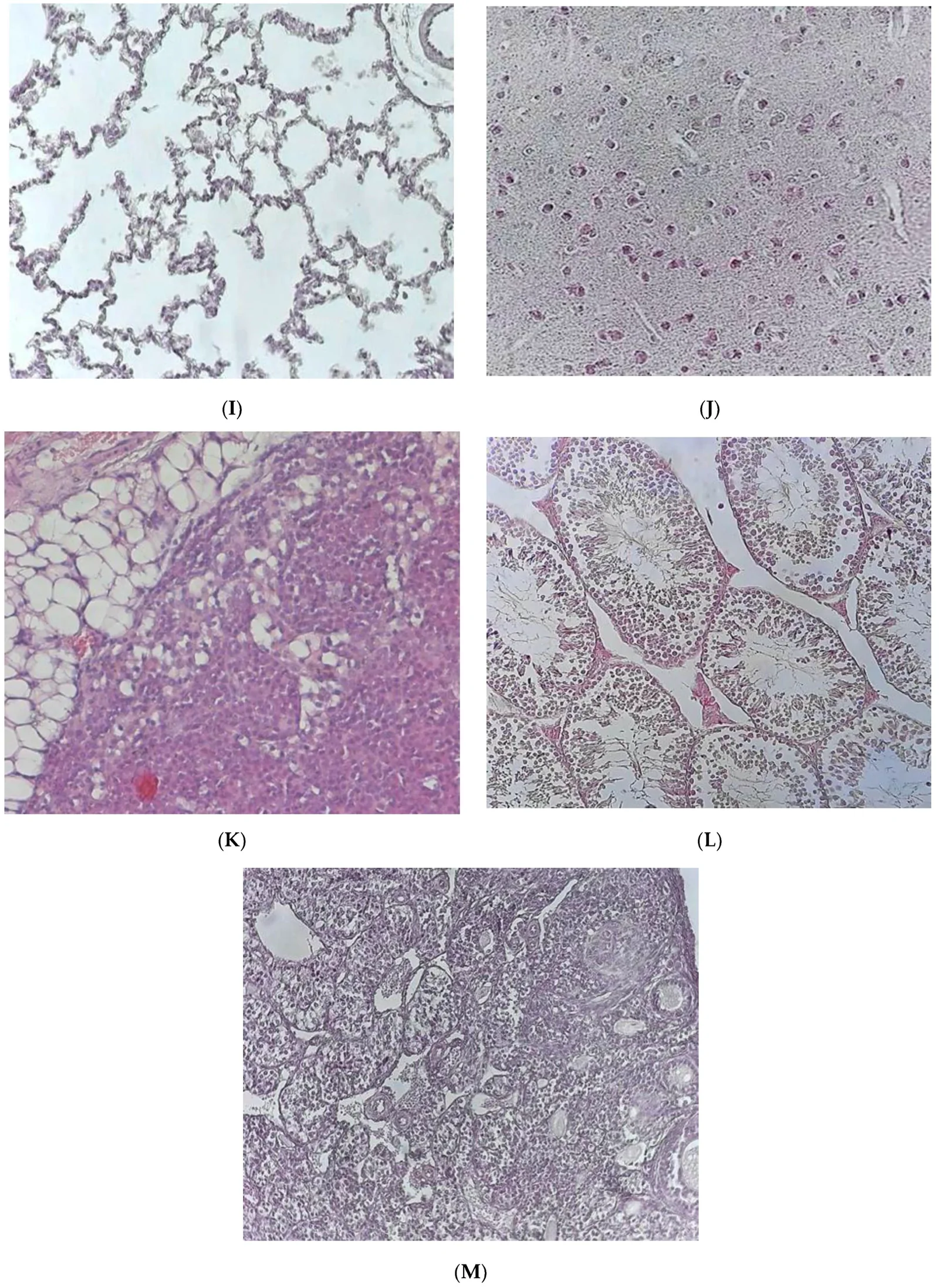
Representative photographs of histopreparations of the internal organs of mice treated with the agent at a dose of 2500 mg/kg for 60 days. Hematoxylin and eosin (H&E) staining. (**A**) Liver (×400); (**B**) kidney (×400); (**C**) spleen (×400); (**D**) heart (×200); (**E**) thymus (×200); (**F**) stomach (×200); (**G**) intestine (×400); (**H**) adrenal gland (all layers; ×200); (**I**) lungs (×400); (**J**) brain (×200); (**K**) lymph node (×400); (**L**) testis (×200); (**M**) ovary (×400).

Figure 1 show representative photographs of histopreparations of the internal organs of the mice treated with the agent at a dose of 2500 mg/kg for 60 days.

The histological structure of the organs of mice receiving the maximum dosage of 2500.0 mg/kg was without pathological changes. Taken together, the data obtained indicate the preservation of normal histoarchitectonics of organs in animals treated with the agent, which indicates the absence of a toxic effect of the agent in the studied doses.

## 4. Discussion

### 4.1. Clinical Signs and General Health Status of Animals

In this study, the safety profile of a medicinal agent based on an association of probiotic lactic acid bacteria, *Lacticaseibacillus rhamnosus* 7n, *Lacticaseibacillus paracasei* B-3/43, and *Lacticaseibacillus casei* 14d/13, combined with *Glycyrrhiza glabra* extract, was assessed following repeated 60-day oral administration. The agent was administered orally for 60 days at doses of 25.0, 250.0 and 2500.0 mg/kg to laboratory mice.

The choice of the composition of the investigated agent was based on a number of our previous studies. Alcoholic licorice root extract showed the greatest inhibitory activity against *Mycobacterium tuberculosis* B5 test culture among the other studied medicinal plants, when exposed to which the growth suppression zone of the test culture was 32 mm [32].

A study of the antimicrobial activity of 11 samples of a probiotic medicinal agent against a clinical strain of *Mycobacterium tuberculosis* was conducted in BioVet Scientific and Production Center LLP (Almaty, Kazakhstan). In the course of this work, eight variants of combined drug formulations and three monocultures of lactic acid bacteria were studied (№1 (*Lacticaseibacillus paracasei* 2B/A-6, *Lacticaseibacillus paracasei* 127/A-4, *Lentilactobacillus farraginis* 27w/60; №2 (*Lacticaseibacillus rhamnosus* 7n, *Lacticaseibacillus rhamnosus paracasei* B-3/43, *Lacticaseibacillus casei* 14d/13; №3 (*Lacticaseibacillus paracasei* 27/A-4, *Lacticaseibacillus paracasei* 2B/A-6, *Lentilactobacillus farraginis* 27w/60); №4 (*Lacticaseibacillus paracasei* 22, *Lentilactobacillus farraginis* 98, *Lentilactobacillus farraginis* 27w/84); №5 (*Lacticaseibacillus rhamnosus* 7n, *Lacticaseibacillus paracasei* B-3/43, *Lacticaseibacillus casei* 14d/13 + 1% licorice extract), №6 (*Lacticaseibacillus paracasei* 22, *Lentilactobacillus farraginis* 98, *Lentilactobacillus farraginis* 27w/84 + 1% licorice extract), №7 (*Lacticaseibacillus rhamnosus* 7n, *Lacticaseibacillus rhamnosus paracasei* Б-3/43, *Lacticaseibacillus casei* 14д/13 + 2% licorice extract), №8 (*Lacticaseibacillus paracasei* 22, *Lentilactobacillus farraginis* 98, *Lentilactobacillus farraginis* 27w/84 + 2% licorice extract), №9 (*Lacticaseibacillus rhamnosus* 7n), №10 (*Lacticaseibacillus paracasei* B-3/43), №11 (*Lacticaseibacillus casei* 14d/13)). The results showed that sample No. 5, including *Lacticaseibacillus rhamnosus* 7n, *Lacticaseibacillus paracasei* B-3/43, *Lacticaseibacillus casei* 14d/13 and 1% licorice extract (*Glycyrrhiza glabra*), retained inhibitory activity at the maximum studied dilution of 1:8, which indicates the prospects of this combination for further study. These results served as the basis for the selection of *Glycyrrhiza glabra* extract and the association of three LAB strains. It is assumed that their combination can complement each other’s action; however, the potential synergistic interactions between laboratory strains and licorice extract require further study [33].

Also, earlier studies were conducted to determine the acute and subacute toxicity of this drug. In this study, acute toxicity was assessed with oral single administration of drug solutions to mice at doses of 25 mg/kg, 250 mg/kg, 2500 mg/kg and 5000 mg/kg and to rats at doses of 27.8 mg/kg, 278.0 mg/kg, 1111.2 mg/kg and 2222.4 mg/kg body weight, and when administered intraperitoneally to the mice at doses of 2.5 mg/kg, 25.0 mg/kg, 250.0 mg/kg, 2500 mg/kg and to the rats at doses of 2.8 mg/kg, 27.8 mg/kg, 278.0 mg/kg, 555.6 mg kg weight. The animals were monitored for signs of acute toxicity for 14 days. Subacute toxicity was assessed by oral administration of doses of 25.0 mg/kg, 250.0 mg/kg and 2500.0 mg/kg to the mice and was monitored for 28 days. As a result, it was found that this drug has no toxic effect on the body and has a favorable safety profile without signs of toxicity. The present study expands on these findings by evaluating its safety after repeated oral administration for 60 days at similar dosages. Considered together, these studies contribute to a broader understanding of the safety profile of the developed drug under various exposure conditions [34].

In general, based on the results of this observation, it can be said that taking the investigational agent within 60 days was well tolerated, and during the experimental period there were no deaths, atypical clinical signs or behavioral changes. The animals maintained normal activity and appearance throughout the study, indicating that there were no visible signs of toxicity when taking the investigational agent.

These results are consistent with the available data from preclinical studies on the safety of probiotic lactic acid bacteria. For example, repeated oral administration of the lactic acid bacterium *Pediococcus acidilactici* J9 strain to mice did not lead to mortality, adverse changes in body weight, and food intake, and no hematological or biochemical abnormal changes were observed [35]. Another study presents data from the *Lactobacillus fermentum* strain MTCC-5898, which was administered orally to mice for 28 days in three doses. Similarly, this strain did not have an adverse effect on the overall health of the studied animals [36].

Since this study was conducted on animals, the results cannot be directly applied to humans. Further research is needed to assess the safety of the drug for humans and evaluate its effects with longer-term use in order to better understand the long-term safety profile and identify potential delayed or cumulative side effects.

### 4.2. Effects on Body Weight and General Physical Condition

During the entire study and recovery period, the animals showed no general health or behavior disorders, including uncoordinated movements, lacrimation, increased salivation or foaming, diarrhea, vomiting, piloerection, crowding of animals, prostration, tremor, difficulty breathing, hunched posture, decreased response to external stimuli, refusal of food, pallor of mucous membrane skin changes, seizures, and decreased skeletal muscle tone. All animals maintained a satisfactory general condition throughout the study. On daily examination, normal motor activity, an adequate response to grabbing, touching, visual and auditory stimuli, as well as a sustained response to tail pinching were noted. The results obtained indicate the absence of clinical signs of systemic toxicity and negative effects of the studied agent on the functional state of the central nervous system and behavioral reactions of animals with prolonged oral administration.

A comparative analysis of body weight dynamics showed that in males and females who received the studied agent at doses of 25.0, 250.0 and 2500.0 mg/kg, body weight changes were comparable with those in the control group. There was no dose-dependent decrease in body weight gain or decrease in animal weight. Slight fluctuations in the body weight of individual animals were of an individual nature and were within the limits of physiological variability. The results obtained indicate that there is no negative effect of the studied agent on the growth, development and general physiological condition of animals with repeated oral administration.

The data obtained are consistent with the published safety results of individual components of the medicinal agent. For example, the prolonged oral administration of *Lactobacillus* spp did not adversely affect the somatic signs and body weight gain, as well as the mass of organs and their relative parameters in mice [37]. In a study of the subchronic toxicity of licorice root extract (*Glycyrrhiza glabra*), no significant changes in body weight or mass of internal organs were found in mice, even after prolonged oral exposure. Taken together, these results confirm the absence of signs of pronounced systemic toxicity at the level of somatic and morphometric parameters of the studied agent [38].

### 4.3. Effects on Feed Intake 

Feed intake and nutritional status of animals in all experimental groups remained comparable with those of the control group throughout the study period.

The study by J. S. Zhou and co-authors investigated the safety of three strains of probiotic bacteria—*Lactobacillus rhamnosus* HN001 (DR20), *L. acidophilus* HN017 and *Bifidobacterium lactis* HN019 (DR10)—in BALB/c mice. The animals were orally injected with the studied strains or the reference strain *L. acidophilus* LA-1 at doses of 2.5 × 10^9^, 5 × 101^0^ and 2.5 × 10^12^ CFU/kg body weight per day for four weeks. During this period, the intake of feed and water, as well as the dynamics of body weight, were monitored. The authors did not note any adverse changes in the general condition, hematological and biochemical parameters of the animals [39].

The study by R. M. Bhide and co-authors evaluated the safety of standardized licorice extract GutGard. To assess acute toxicity, no deaths or clinical signs of toxicity were observed with a single oral dose of 5000 mg/kg during a 14-day follow-up period. In a 90-day study of repeated oral administration of extract doses of 250, 500 and 1000 mg/kg to rats, no adverse clinical signs associated with the drug were also detected. The animals’ body weight gain and feed intake were comparable to those of the control groups [40].

These studies indicate that monitoring of feed intake and body weight dynamics are important indicators in assessing the safety of probiotic bacteria and licorice extract. They also serve as an additional literary context for interpreting the data from our study, although differences in lactic acid bacteria strains, drug composition, doses, and duration of exposure do not directly equate their results with the safety of the studied drug.

### 4.4. Interpretation of Macroscopic Findings

After 60 days of oral administration of the investigational agent, a macroscopic analysis of the internal organs of the animals was performed, as a result of which no visible pathological changes were detected. All the organs studied retained the shape, size, color, and consistency characteristic of intact animals. There were no signs of edema, hemorrhage, hyperemia, necrotic changes or other damage. These data are an additional sign of the absence of a pronounced toxic effect of the agent under study on internal organs with the chosen duration and regimen of administration.

### 4.5. Interpretation of Hematological Findings

According to the hematology study, after 60 days of oral administration of the investigational medicinal agent in three doses, most blood parameters, including the number of red blood cells, hemoglobin level, hematocrit, leukocytes, platelets and leukocyte formula, remained comparable with the values of the control group. A moderate increase in the number of red blood cells in males at a dose of 2500 mg/kg and in females at a dose of 25 mg/kg was not accompanied by an increase in hemoglobin and hematocrit; therefore, it did not indicate the development of pathological changes. Also, a slight increase in the number of white blood cells in females at a dose of 2500 mg/kg was not accompanied by neutrophilic leukocytosis and a change in the leukocyte formula, which allows us to consider this deviation as an option. The results obtained during this work are consistent with the data of toxicological studies of probiotic lactic acid bacteria: after 28 days of oral administration of *Lactobacillus helveticus* KLDS1.8701 at a dose of 6 × 10^10^ CFU in rats we did not observe significant changes in the number of red blood cells, leukocytes, and hemoglobin, platelet counts or leukocyte counts compared to the those of the control [41].

### 4.6. Interpretation of Biochemical Findings

The results of the biochemical study indicate that repeated administration of the probiotic in doses of 25, 250 and 2500 mg/kg does not adversely affect the functional state of the liver, kidneys and the main types of metabolism in mice of both sexes. The revealed differences between individual animals are not dose-dependent, are within the limits of physiological variability and are not toxicologically significant.

### 4.7. Interpretation of Urinalysis Findings

After the end of the time chosen for the study, no pathological changes in the urine composition were detected. The main physico-chemical and microscopic parameters remained comparable with those of the control group, which indicates the absence of signs of impaired urinary system function under the selected experimental conditions.

### 4.8. Interpretation of Histological Findings

Histological examination was performed only on animals receiving the maximum studied dosage of the agent—2500 mg/kg. Microscopic examination of the tissues of internal organs did not reveal any morphological changes indicating the toxic effect of the agent under study. The histological structure of the organs remained comparable to that of the control animals, with no signs of inflammation, dystrophic, necrotic, or other pathological changes. The analysis of the results obtained and their comparison with the literature data serve as the basis for approving the safety of the drug in the studied doses.

## 5. Conclusions

Thus, in the course of this study, a favorable safety profile of the medicinal probiotic was proven based on the association of probiotic lactic acid bacteria *Lacticaseibacillus rhamnosus* 7n, *Lacticaseibacillus parasasei* B-3/43, *Lacticaseibacillus casei* 14d/13 and licorice extract *Glycyrrhiza glabra*. The use of the investigational agent for 60 days was not accompanied by the detection of signs of general toxicity, significant hematological, biochemical and physiological disorders, changes in urine composition, as well as macroscopic and histopathological changes in internal organs in experimental animals.

The results obtained are consistent with the data from similar studies. Therefore, based on the results of preclinical trials, in particular on the absence of toxicity, in the future it would be possible to conduct clinical trials and use this medicinal probiotic as a therapeutic and prophylactic drug in the complex therapy of tuberculosis.

## Figures and Tables

**Table 1:** Body weight changes in male mice after oral administration of a medicinal probiotic agent in various doses during the observation period (days 1–30), (M ± SD).

№ Group	Doze	Average Weight of Animals, g, M ± SD				
		Before Experiment	After 7 Days	After 14 Days	After 21 Days	After 28 Days
1	25.0	21.5 ± 0.868	24.4 ± 0.613	26.2 ± 0.814	27.8 ± 1.322	30.5 ± 1.720
3	250.0	21.9 ± 1.516	23.5 ± 1.650	26.3 ± 1.494	28.9 ± 1.414	31.7 ± 1.688
5	2500.0	21.6 ± 1.160	23.5 ± 1.225	25.6 ± 1.223	27.4 ± 1.260	30.2 ± 1.194
7	Saline solution	21.9 ± 1.517	23.7 ± 1.190	25.8 ± 0.698	27.3 ± 0.786	30.0 ± 0.893
9	Intact	21.9 ± 1.258	24.6 ± 0.912	26.8 ± 1.027	28.5 ±1.403	28.9 ± 1.361

**Table 2:** Body weight changes in male mice after oral administration of a medicinal probiotic agent in various doses during the observation period (31–60 days), (M ± SD).

№ Group	Doze	Average Weight of Animals, g, M ± SD				
		After 35 Days	After 42 Days	After 49 Days	After 56 Days	End of the Experiment
1	25.0	33.2 ± 1.344	34.1 ± 1.420	34.8 ± 1.268	35.6 ± 1.388	36.3 ± 1.447
3	250.0	34.2 ± 1.378	34.6 ± 0.906	35.2 ± 0.859	36.0 ± 0.952	36.5 ± 0.895
5	2500.0	32.0 ± 1.617	32.6 ± 1.568	33.1 ± 1.564	33.7 ± 1.485	34.4 ± 1.521
7	Saline solution	31.9 ± 1.156	32.6 ± 1.124	33.2 ± 1.161	33.8 ± 0.998	34.6 ± 1.003
9	Intact	30.0 ± 1.861	30.6 ± 2.066	31.2 ± 1.996	31.7 ± 1.737	32.4 ± 1.679

**Table 3:** Body weight changes in female mice after oral administration of a medicinal probiotic agent in various doses during the observation period (days 1–30), (M ± SD).

№ Group	Doze	Average Weight of Animals, g, M ± SD				
		Before Experiment	After 7 Days	After 14 Days	After 21 Days	After 28 Days
2	25.0	21.2 ± 1.315	22.5 ± 1.540	23.7 ± 1.656	25.2 ± 1.908	28.6 ± 1.763
4	250.0	22.0 ± 1.540	23.6 ± 1.329	25.3 ± 0.983	27.3 ± 0.606	29.3 ± 0.560
6	2500.0	21.7 ± 0.804	23.7 ± 0.738	26.0 ± 1.016	27.5 ±0.864	29.4 ± 0.987
8	Saline solution	21.4 ± 0.821	23.1 ± 1.131	25.2 ± 0.780	27.0 ± 0.976	29.5 ± 0.956
10	Intact	21.6 ± 1.021	23.2 ± 1.022	25.2 ± 1.188	27.1 ± 1.343	29.2 ± 1.458

**Table 4:** Body weight changes in female mice after oral administration of a medicinal probiotic agent in various doses during the observation period (31–60 days), (M ± SD).

№ Group	Doze	Average Weight of Animals, g, M ± SD				
		After 35 Days	After 42 Days	After 49 Days	After 56 Days	End of the Experiment
2	25.0	31.1 ± 1.221	31.5 ± 1.182	32.1 ± 1.113	32.6 ± 1.123	33.4 ± 1.088
4	250.0	31.3 ± 1.052	31.8 ± 1.011	32.3 ± 0.983	32.9 ± 0.986	33.5 ± 1.008
6	2500.0	31.0 ± 1.595	31.4 ± 1.505	32.0 ± 1.496	32.6 ± 1.488	33.2 ± 1.471
8	Saline solution	31.0 ± 0.932	31.5 ± 0.923	32.1 ± 0.923	32.7 ± 0.935	33.3 ± 0.972
10	Intact	32.0 ± 1.119	32.5 ± 1.106	33.1 ± 1.126	33.7 ± 1.120	34.3 ± 1.155

**Table 5:** Body weight dynamics and body weight gain coefficient in male mice with 60-day oral administration of the studied agent.

Group	*n*	Mass at the Beginning, g	Mass at the End, g	Coefficient of Increase, %
25 mg/kg	10 ♂	21.5 ± 0.868	36.3 ± 1.447	68.8
250 mg/kg	10 ♂	21.9 ± 1.516	36.5 ± 0.895	66.6
2500 mg/kg	10 ♂	21.6 ± 1.160	34.4 ± 1.521	59.2
Saline solution	10 ♂	21.9 ± 1.517	34.6 ± 1.003	57.9
Intact	10 ♂	21.9 ± 1.258	32.4 ± 1.679	47.9

**Table 6:** Body weight dynamics and body weight gain coefficient in female mice with 60-day oral administration of the studied agent.

Group	*n*	Mass at the Beginning, g	Mass at the End, g	Coefficient of Increase, %
25 mg/kg	10 ♂	21.2 ± 1.315	33.4 ± 1.088	57.5
250 mg/kg	10 ♂	22.0 ± 1.540	33.5 ± 1.008	52.2
2500 mg/kg	10 ♂	21.7 ± 0.804	33.2 ± 1.471	52.9
Saline solution	10 ♂	21.4 ± 0.821	33.3 ± 0.972	55.6
Intact	10 ♂	21.6 ± 1.021	34.3 ± 1.155	58.7

**Table 7:** Average values of animal weight by group.

Group	The Average Value	*n*
25 mg/kg	21.31	2
250 mg/kg	21.93	2
2500 mg/kg	21.66	2
Saline solution	21.65	2
Intact	21.78	2

**Table 8:** Results of a one-factor analysis of variance.

The Source of the Variation	SS	df	MS	F; *p*
Between groups	0.4172	4	0.1043	F(4, 5) = 1.907; *p* = 0.2476
Within groups (remainder)	0.2735	5	0.05469	—
Total	0.6906	9	—	—

Note: SS—the sum of squares; df—the number of degrees of freedom; MS—the average square.

**Table 9:** Pairwise comparisons of groups by the Sidak criterion.

Group Comparison	Difference in Averages	95% Confidence Interval (CI) for the Difference	Adjusted *p*	Conclusion
25 mg/kg vs. 250 mg/kg	−0.620	−1.703…0.4628	0.3415	Not significant
25 mg/kg vs. 2500 mg/kg	−0.350	−1.433…0.7328	0.8576	Not significant
25 mg/kg vs. Control	−0.340	−1.423…0.7428	0.8742	Not significant
25 mg/kg vs. Intact control	−0.465	−1.548…0.6178	0.6258	Not significant
250 mg/kg vs. 2500 mg/kg	0.70	−0.8128…1.353	0.9599	Not significant
250 mg/kg vs. Control	0.80	−0.8028…1.363	0.9511	Not significant
250 mg/kg vs. Intact control	0.55	−0.9278…1.238	0.9990	Not significant
2500 mg/kg vs. Control	0.010	−1.073…1.093	>0.9999	Not significant
2500 mg/kg vs. Intact control	−0.115	−1.198…0.9678	>0.9999	Not significant

## Data Availability

The data presented in this study are available from the corresponding author upon reasonable request.

## Supplementary Materials

The following supporting information can be downloaded at: https://www.mdpi.com/article/10.3390/biomedicines14102364/s1, File S1. List of Standard Operating Procedures Applicable to the Animal Study.

## Abbreviations

The following abbreviations are used in this manuscript:

LAB	Lactic Acid Bacteria
GLP	Good Laboratory Practice
CFU	Colony-forming units
RCM	Republican Collection of Microorganisms
MRS	de Man, Rogosa and Sharpe medium
RBC	Red Blood Cells
HGB	Hemoglobin
HCT	Hematocrit
WBC	White Blood Cells
PLT	Platelets
SD	Standard deviation
AST	Aspartate transaminase
ALT	alanine transaminase
