# Supplementary material for: Assessment of the Safety of a Combined Medicinal Agent Based on Probiotic Lactic Acid Bacteria and the Natural Component Glycyrrhiza glabra
Source: Biomedicines. 2026 Oct 16;14(10):2364. doi: 10.3390/biomedicines14102364 (PMC13701990; doi:10.3390/biomedicines14102364)
Supplement: Supplementary file 1 [file biomedicines-14-02364-s001.zip › biomedicines-4613070-supplementary.pdf]

List of Standard Operating Procedures Applicable to the Animal Study

The animal study was conducted in accordance with the applicable institutional Standard Operating Procedures (SOPs). The following SOPs were applied during animal care, experimental procedures, monitoring, administration of the test item, euthanasia, necropsy, histological examination, and study documentation.

SOP-PHT-001 — Laboratory Animal Care
SOP-PHT-002 — Cleaning and Disinfection of Laboratory Rooms and Animal Facility
SOP-PHT-003 — Animal Randomization in Experiments
SOP-PHT-004 — Animal Marking
SOP-PHT-007 — Cleaning and Sterilization of Laboratory Equipment
SOP-PHT-009 — Administration of Test Items
SOP-PHT-010 — Temperature and Humidity Monitoring
SOP-PHT-011 — Veterinary Care
SOP-PHT-016 — Rodent Analgesia, Anesthesia and Euthanasia
SOP-PHT-019 — Macroscopic Examination of Internal Organs of Experimental Animals
SOP-PHT-020 — Preparation of Histological Sections
SOP-PHT-021 — Animal Observation
SOP-PHT-022 — Storage of Feed and Feeding Laboratory Animals
SOP-GE-021 — Medical Waste: Waste Management Programme
SOP-D-002 — Study Plans
SOP-D-003 — Raw Data
SOP-D-004 — Study Reports
SOP-TI-001 — Handling of Test Items
SOP-EQ-026 — Water Distiller Operation

All procedures were performed in accordance with the applicable institutional SOPs and established laboratory procedures.
